# Regulation of mammalian horizontal gene transfer by apoptotic DNA fragmentation

**DOI:** 10.1038/sj.bjc.6603484

**Published:** 2006-12-05

**Authors:** B Yan, H Wang, F Li, C-Y Li

**Affiliations:** 1 Department of Radiation Oncology, Duke University Medical Center, Durham, NC 27710, USA; 2 Department of Medicine, Duke University Medical Center, Durham, NC 27710, USA; 3 Department of Radiation Oncology, University of Colorado Health Sciences Center, Aurora, CO 80045, USA

**Keywords:** horizontal gene transfer, apoptosis, DNA fragmentation factor, caspase activated DNase

## Abstract

Previously it was shown that horizontal DNA transfer between mammalian cells can occur through the uptake of apoptotic bodies, where genes from the apoptotic cells were transferred to neighbouring cells phagocytosing the apoptotic bodies. The regulation of this process is poorly understood. It was shown that the ability of cells as recipient of horizontally transferred DNA was enhanced by deficiency of p53 or p21. However, little is known with regard to the regulation of DNA from donor apoptotic cells. Here we report that the DNA fragmentation factor/caspase-activated DNase (DFF/CAD), which is the endonuclease responsible for DNA fragmentation during apoptosis, plays a significant role in regulation of horizontal DNA transfer. Cells with inhibited DFF/CAD function are poor donors for horizontal gene transfer (HGT) while their ability of being recipients of HGT is not affected.

DNA fragmentation is a biochemical hallmark of apoptosis. Apoptotic DNA fragmentation involves the incision of chromosomal DNA in-between nucleosomes. The nuclease responsible for apoptotic DNA fragmentation is caspase-activated DNase (CAD), also called DNA fragmentation factor (DFF) (Liu *et al*, 1997; Enari *et al*, 1998; Lechardeur *et al*, 2000). Caspase-activated DNase is normally associated with its inhibitor as well as chaperon, inhibitor of CAD (ICAD). During apoptosis, the activated caspase 3 cleaves ICAD and releases CAD to digest chromosomal DNA. Apoptotic DNA fragmentation is a conserved process across many species in evolution. However, its function is not completely understood. It is not essential for embryonic development as the CAD knockout mice show no obvious abnormality (Zhang *et al*, 1998; Kawane *et al*, 2003). One group reported that it probably play a role in learning and memory (Slane *et al*, 2000). We have shown previously that CAD gene is important for tumour suppression, especially when the organism is exposed to DNA-damaging agents (Yan *et al*, 2006a).

Horizontal gene transfer (HGT) is the collective name for processes that permit the exchange of DNA among individuals of the same or different species. It plays an important role in evolution of bacteria and fungi, for instance, in generation of drug-resistance and adaptation to new environments. In bacteria, genes can be transferred by means of transformation, conjugation, and transduction (Jain *et al*, 1999, 2002). Horizontal transfer of genes can also occur between somatic cells of mammalian origin by uptake of apoptotic bodies (Holmgren *et al*, 2002). This is an important discovery as it is generally assumed that DNA from apoptotic cells is degraded and the encoded genetic information is lost upon completion of the apoptotic process. Several reports have shown that mammalian HGT can occur from apoptotic cells to surrounding live cells via phagocytosis (Holmgren *et al*, 1999; Bergsmedh *et al*, 2001). Moreover, the transfer of DNA can be a very efficient process. By fluorescence *in situ* hybridisation, DNA of apoptotic cells was found in nuclei of as much as 15% of phagocytosing (recipient) cells (Holmgren *et al*, 1999).

The molecular mechanism for the regulation of the HGT process is only partially understood. Horizontal gene transfer involves donor cells (apoptotic cells) and recipient cells (phagocytosing cells). Transfer of apoptotic DNA to wild-type mouse embryonic fibroblast cells (MEF) trigger cell cycle arrest or senescence in a p53-dependent pathway (Holmgren *et al*, 1999; Bergsmedh *et al*, 2001). P53 or p21-deficient cells are able to receive apoptotic DNA and continue to proliferate (Bergsmedh *et al*, 2002). However, regulation of HGT in the donor cells has not been studied. In this report, we showed that internucleosomal digestion of chromosomes by CAD facilitates the transfer of gene from apoptotic cells to phagocytosing cells.

## MATERIALS AND METHODS

### Cells

TK6 lymphoblastoid cells were obtained from ATCC (Manassas, VA, USA) and cultured in RPMI-1640 supplemented with 10% equine serum. L929 cells were maintained in DMEM medium supplemented with 10% equine serum. The wild-type and CAD-null MEFs were isolated from embryos as described previously (Yan *et al*, 2006a). P53-null MEFs were obtained from Dr Patricia Hardenberg of Duke University (Durham, NC, USA). All MEFs were cultured in DMEM with 10% foetal bovine serum.

### Plasmid construction and gene transduction

A modified ICAD gene was constructed and stably transduced into TK6 and L929 cells as described previously (Yan *et al*, 2006a).

### Western blotting

Cells were collected, washed in PBS, and lysed in 1% Triton lysis buffer (Yan *et al*, 2003). Samples were denatured at 100°C for 5 min. Equal amount of total protein were loaded to each well for electrophoresis in 10% SDS polyacrylamide gels and then transferred to polyvinylidene fluoride microporous membranes (Millipore Corporation, Billerica, MA, USA). Membranes were then incubated with primary antibody followed by incubation with horseradish peroxidase-linked secondary antibodies. Antibody–antigen complexes were detected using chemiluminescence (Pierce Biotechnology, Rockford, IL, USA). The primary antibody used is an anti-HA tag antibody (Roche Diagnostics, Pleasanton, CA, USA).

### DNA ladder assay

Thirty-six hours after treatment of 5 × 10^6^ cells with 100 ng ml^−1^ TNF*α*, cells were detached and washed with PBS. The collected cells were lysed in 100 *μ*l lysis buffer containing 10 mM Tris, 6 mM EDTA, 0.5% SDS (W/V), pH 8.0, and 0.5 mg ml^−1^ proteinase K at 55°C overnight. After phenol : chloroform and chloroform extractions, DNA were precipitated by adding two volumes of ethanol, 1/10 volume of 3 M sodium acetate, and 1/100 volume of 1 M magnesium chloride and being incubated at −20°C overnight. DNA pellet was washed with 70% ethanol and dissolved in TE buffer. RNA was removed by incubating with 0.5 mg ml^−1^ RNAse at 37°C for 30 min. The purified DNA were then subjected to elctrophoresis in a 2% agarose gel and visualised under UV light after staining with ethidium bromide.

### Apoptosis evaluation by annexin V staining

To quantify the amount of cellular apoptosis in mutant ICAD (mICAD)-transfected cells, a method based on the staining of externalised phosphotidyl serine by annexin V staining was adopted. A commercially available annexin V staining kit (Clontech, Palo Alto, CA, USA) was used. Control and radiation treated cells were stained according to the manufacturer's instruction. Double annexin V/propidium iodide staining was used to distinguish cells that were undergoing early *vs* late apoptosis. The fraction of cells with positive annexin V staining was defined as cells undergoing apoptosis. Quantification of annexin V staining was carried out by the Duke University Cancer Center Flow Cytometry Facility.

### Gene transfer by co-cultivation experiments

About 10^7^ donor cells were irradiated with large doses *γ*-ray that is enough to kill all the cells, specifically 20 Gy for TK6 cells and 200 Gy for L929 cells. Immediately (for TK6 cells) or 3 days (for L929 cells, in PBS) after radiation, the apoptotic donor cells were co-cultured with 1–2 × 10^6^ recipient cells in 10 cm Petri dishes for 3 days and then the medium were changed every 3–5 days and the cells were grown in the presence of puromycin (5 *μ*g ml^−1^). About 2–3 weeks after puromycin-selection, the colonies were fixed and stained with 0.5% crystal violet dissolved in 80% methanol.

### Polymerase chain reaction analysis

Genomic DNA was isolated from various clones of puromycin-resistant cells using DNeasy Tissue Kit (QIAGEN). Polymerase chain reaction (PCR) was performed with specific primers for puromycin-resistant gene (puro^r^). Sense primer is 5′-GTCACCGAGCTGCAAGAACTC-3′, antisense primer is 5′-GTCCTTCGGGCACTCGAC-3′. The size of amplified product is 426 bp.

## RESULTS

### Establishing cell lines with inhibition of DNA fragmentation by mutant inhibitor of caspase activated DNase

**Figure 1:**
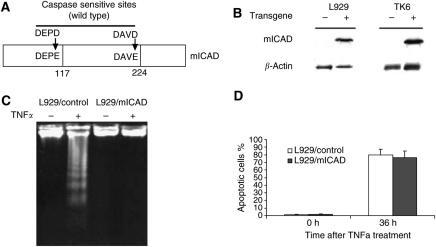
Generation of genetically modified cell lines with significantly inhibited DNA fragmentation. (**A**) The structure of the modified ICAD/DFF45 protein. Polymerase chain reaction-based approaches were used to generate two point mutations in the two caspase-sensitive sites that are essential for the activation of the nuclease activities of CAD/DFF40. The mutations lead to amino acids changes that render the mICAD protein resistant to caspase cleavage. (**B**) Western blot analysis of mICAD protein expression in cells that have been stably transduced with a mICAD gene. An antibody (from Roche Molecular Biology) against the HA tag that was engineered into the 3′ end of the mICAD gene was used so that only the modified ICAD gene is detected. It is clear that only those cells that were transduced with the mICAD gene express the mutant form of the ICAD protein. (**C**) Mutant ICAD inhibited DNA ladder formation in apoptotic cells. DNAs were extracted from apoptotic L929 cells 36 h after TNF*α* treatment and the DNA ladder was separated by electrophoresis. (**D**) Apoptosis rate of cells after TNF*α* treatment evaluated by annexin V/propidium iodide staining.

Inhibitor of CAD is a chaperon as well as an inhibitor of CAD. Inhibitor of CAD is normally complexed with CAD and inhibits its DNase activity. When apoptosis is induced, ICAD is cleaved by caspase and CAD is released to carry out DNA fragmentation in the nucleus. There are two caspase cleavage sites in ICAD, D117, and D224. We engineered a mICAD according to a published method by introducing point mutations at the two cleavage sites, D117E and D224E (Figure 1A). Mutant ICAD is resistant to caspase cleavage and inhibits DNA fragmentation during apoptosis in both human and mouse cells (Sakahira *et al*, 1998). We established two cell lines expressing mICAD, TK6, and L929, as shown in Figure 1B. Inhibition of DNA fragmentation by mICAD was also confirmed by DNA ladder assay (Figure 1) and DNA histogram in these stable cells (data not shown). Reduced DNA fragmentation in mICAD cells was not due to decreased apoptosis because the control and mICAD-expressing cells underwent apoptosis at about the same rate as determined by annexin V staining, which evaluates a maker that is independent of DNA fragmentation (Figure 1D). Therefore, expression of mICAD inhibits apoptotic DNA fragmentation significantly without affecting apoptotic cell death significantly.

### Regulation of horizontal gene transfer in the donor cells

**Figure 2:**
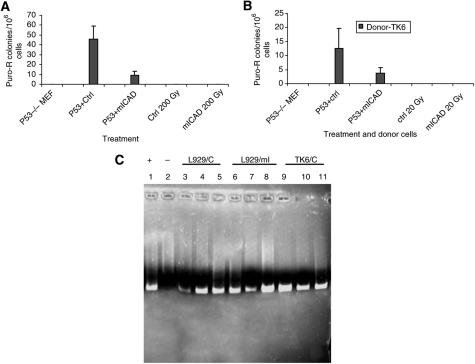
Inhibition of DNA fragmentation in donor cells led to decreased gene transfer. About 10^7^ apoptotic control and mICAD-expressing donor cells containing puro^r^ gene were added to 2 × 10^6^ p53−/− cells and co-cultured for 3 days. Then MEF cells underwent puromycin selection for 2 weeks and the frequency of puro^r^ gene transfer was calculated by counting the surviving colonies. About 10^7^ apoptotic donor cells, 2 × 10^6^ p53−/− MEF cells alone (without co-culture with apoptotic donors) were also plated in 10 cm dishes and underwent puromycin selection as controls. The donor cells are L929 (**A**) and TK6 (**B**) cells. (**C**) Confirmation of transferred gene in puromycin-resistant MEFs by PCR.

Horizontal gene transfer between somatic cells of mammalian is achieved by uptake of apoptotic bodies by surrounding cells or phagocytes. The apoptotic cells that provide the transferred DNA are called donor cells whereas the neighbouring live cells that phagocytose the apoptotic bodies are recipient cells. Recently, it was reported that the p53 and p21 gene status in the recipient cells plays significant roles in regulating HGT (Bergsmedh *et al*, 2002). We decided to examine the effect of DFF/CAD on this process because we reasoned that the enzyme that digests DNA in the apoptotic cells is very likely to play some roles in HGT. In the initial experiment, we used as donor cells both the control and mICAD-expressing cells that have an exogenous puro^r^ integrated into their genomes. We induced apoptosis of donor cells by irradiation and then co-cultured the irradiated apoptotic cells with p53−/− MEFs to allow phagocytosis, integration, and expression of the transferred gene. Mouse embryonic fibroblasts do not have puro^r^ gene and are sensitive to puromycin. Only those MEF cells that successfully obtained and express the puro^r^ gene will gain resistance to puromycin. We found that MEFs cultured with control donor cells formed much more puromycin-resistant colonies than those cultured with the mICAD-expressing donor cells. Therefore the donor cells with normal CAD function led to higher frequency of HGT than those transfected with mICAD. This is consistently shown in two different donor cell lines (Figure 2A and B). Polymerase chain reaction using the puro^r^-specific primers was performed to confirm the presence of transferred gene in puromycin-resistant MEF cells (Figure 2C).

### Caspase-activated DNase does not regulate horizontal gene transfer by affecting recipient cells

**Figure 3:**
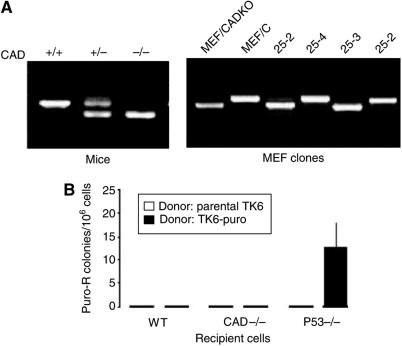
Lack of effect on HGT by CAD in the recipient cells. (**A**) Genotyping of CAD−/− mice and isolated MEF clones. Wild type produces a single upper band, heterozygote produces two bands, and the homozygous CAD knockout has a single lower band. (**B**) Transfer of puro^r^ gene to CAD−/− MEF cells. Parental TK6 or stable TK6 cells containing puro^r^ gene were induced to undergo apoptosis by radiation. The apoptotic cells were incubated with wild type, CAD−/−, or p53−/− MEFs for 3 days before puromycin selection.

In the second experiments, we evaluated whether the CAD gene status of the recipient cells affects HGT. In order to do this, we isolated MEF cells from wild-type and CAD−/− mice and used them as recipient cells in the puro^r^ gene transfer experiment. Similar to the wild-type MEFs, the CAD−/− MEFs co-cultivated with apoptotic cells containing puro^r^ gene fail to form any colonies under puromycin selection (Figure 3). This result suggests that CAD status in the recipient cells does not regulate HGT.

## DISCUSSION

In this study, we demonstrate a clear effect of apoptotic DNA fragmentation in regulating HGT. An important unanswered question is how does DNA fragmentation facilitate the transfer of DNA by uptake of apoptotic bodies. A rational speculation is that the shorter DNA fragments and the break ends generated from DFF digestion of the genome may facilitate the entry of DNA into recipient cells and integration into host genome.

What is the biological significance of the regulation of horizontal DNA transfer by DFF? At this stage only speculations are available. Especially in prokaryotes, horizontal transfer is considered to be a major contributor to the evolution of genome (Jain *et al*, 1999). This is based on the theory of chimeric evolution, which is based on the assumption of the chimeric origins of eucaryotic genomes. It suggests that HGT is an important evolutionary mechanism in eucaryotes as well as in prokaryotes (Lake *et al*, 1999). Lateral transfer of DNA between eucaryotic cells can be achieved by uptake of apoptotic bodies. Therefore, a potential role of apoptotic DNA fragmentation is the facilitation of horizontal transfer of genes that drives the progress of evolution.

Is there any role for HGT in tumour development? It was shown that horizontal transfer of oncogenes by uptake of apoptotic bodies promotes cellular transformation and tumorigenesis (Bergsmedh *et al*, 2001). In reality, the transferred genes from apoptotic bodies can be either tumour-promoting genes or tumour-suppressor genes. Nevertheless, the cells transferred with oncogenes gain growth advantage and outgrow others. Therefore the transfer of oncogene is manifested whereas that of tumour suppressor gene is not.

However, the overall effect of DFF on tumour is that it suppresses tumour development (Yan *et al*, 2006a, 2006b). This may be due to two reasons. First, the major effect of DNA fragmentation is to ensure the complete destruction of apoptotic cells and therefore oppose tumour development. Inhibition of DNA fragmentation leads to resistance to apoptotic cell death (Zhang *et al*, 1998; Yan *et al*, 2006a). Second, the successful HGT is a relatively low-frequency event. This effect may be overshadowed by DFF's major effect of removing DNA-damaged/mutated cells.
